# Molecular Markers and Antimicrobial Resistance Patterns of Extraintestinal Pathogenic *Escherichia coli* from Camel Calves Including Colistin-Resistant and Hypermucoviscuous Strains

**DOI:** 10.3390/tropicalmed9060123

**Published:** 2024-05-23

**Authors:** Domonkos Sváb, Zoltán Somogyi, István Tóth, Joseph Marina, Shantymol V. Jose, John Jeeba, Anas Safna, Judit Juhász, Péter Nagy, Ahmed Mohamed Taha Abdelnassir, Ahmed Abdelrhman Ismail, László Makrai

**Affiliations:** 1 HUN-REN Veterinary Medical Research Institute, H-1143 Budapest, Hungary; tothistvan3@yahoo.co.uk; 2 Department of Pharmacology and Toxicology, University of Veterinary Medicine, H-1078 Budapest, Hungary; somogyi.zoltan@univet.hu; 3 Central Veterinary Research Laboratory, Dubai P.O. Box 597, United Arab Emirates; mjoseph@cvrl.ae (J.M.); shanty@cvrl.ae (S.V.J.); jeeba@cvrl.ae (J.J.); safna@cvrl.ae (A.S.); 4 Farm and Veterinary Department, Emirates Industry for Camel Milk and Products, Dubai P.O. Box 294236, United Arab Emirates; jutka@camelicious.ae (J.J.); peter@camelicious.ae (P.N.); nataha@camelicious.ae (A.M.T.A.); drabohmaid@hotmail.com (A.A.I.); 5 Autovakcina Ltd., H-1171 Budapest, Hungary; autovakcina@gmail.com

**Keywords:** ExPEC, NTEC, EPEC, camel, septicemia, hypermucoviscosity, pathogenicity islands, antibiotic resistance, multidrug resistance

## Abstract

Extraintestinal pathogenic *Escherichia coli* (ExPEC) strains are capable of causing various systemic infections in both humans and animals. In this study, we isolated and characterized 30 *E. coli* strains from the parenchymatic organs and brains of young (<3 months of age) camel calves which died in septicemia. Six of the strains showed hypermucoviscous phenotype. Based on minimum inhibitory concentration (MIC) values, seven of the strains were potentially multidrug resistant, with two additional showing colistin resistance. Four strains showed mixed pathotypes, as they carried characteristic virulence genes for intestinal pathotypes of *E. coli*: three strains carried *cnf1,* encoding cytotoxic necrotizing factor type 1, the key virulence gene of necrotoxigenic *E. coli* (NTEC), and one carried *eae* encoding intimin, the key virulence gene of enteropathogenic *E. coli* (EPEC). An investigation of the integration sites of pathogenicity islands (PAIs) and the presence of prophage-related sequences showed that the strains carry diverse arrays of mobile genetic elements, which may contribute to their antimicrobial resistance and virulence patterns. Our work is the first to describe ExPEC strains from camels, and points to their veterinary pathogenic as well as zoonotic potential in this important domestic animal.

## 1. Introduction

*Escherichia coli*, besides including numerous intestinal pathotypes, includes several lineages of strains capable of causing extraintestinal disease, collectively referred to as extraintestinal pathogenic *E. coli* (ExPEC) [1]. ExPEC are responsible for a range of urinary tract infections (UTI), in which case they are referred to as uropathogenic *E. coli* (UPEC) [2,3], newborn meningitis (NMEC), and other kinds of sepsis and systemic infections, in humans and animals alike [4].

While intestinal pathotypes of *E. coli* are defined by the carriage of one or few key virulence genes (VGs), ExPEC are more heterogeneous in their virulence apparatus, and it seems that there is no single VG that enables an ExPEC strain to cause a site-specific infection [4]. These infections are more likely to be a multifactorial process, involving fitness genes broadly distributed among commensal *E. coli*, suggesting that there is no strong selective pressure on *E. coli* to become an ExPEC, and that many ExPEC are actually opportunistic pathogens, with no clear distinction between commensal *E. coli* and ExPEC [5].

Another difference between ExPEC and intestinal pathotypes of *E. coli* is that the source of infection is often difficult to identify, as the pattern of infections does not follow the classic ‘outbreak’ scheme [6].

Similarly to intestinal pathogenic *E. coli*, ExPEC are genetically quite heterogeneous, with mobile genetic elements (MGE) forming a significant part of their genome, and playing an important role in the dissemination of VGs and those related to antimicrobial resistance (AMR) [7]. Pathogenicity islands (PAIs) are considered significant MGEs in pathogenic *E. coli*, especially in the case of UPEC, where important VGs are encoded on PAIs, which make up a significant proportion of the whole genome [8,9,10].

Camels are uniquely important domestic animals in many countries of North Africa and the Middle East, and the study of their infectious diseases is, therefore, of utmost importance from the perspective of food safety, public health and economy as well [11]. Various pathotypes of intestinal pathogenic *E. coli*, as well as *E. coli* of unspecified pathotypes carrying ARGs (antimicrobial resistance related genes), especially those encoding extended-spectrum beta-lactamase (ESBL) have been characterized from camels in the past decades [12,13,14,15].

In this study, our aim was the molecular and phenotypic characterization of 30 *E. coli* strains isolated from camel calves which died in sepsis. To our knowledge, no *E. coli* strains isolated from extraintestinal infections in camels have been reported to date; therefore, our findings could enrich our knowledge about the virulence and resistance characteristics, and thus, the zoonotic potential of such strains.

## 2. Materials and Methods

### 2.1. Bacterial Strains, Isolation and Culturing

Strains in the study originated from dead camel calves from two camel farms (F1 and F2) in the United Arab Emirates (UAE), involved in camel milk production. All animals were below three months of age, with some of them being younger than one month at the time of death. Bacteriological samples were taken from parenchymal organs during the post-mortem examination of the corpses transported to the diagnostic laboratory after death. After burning off the surface of the parenchymal organ samples with a heated flat metal instrument (decontamination), sampling was performed through the burnt surface using a sampling cotton swab. These swabs were then directly spotted onto the surface of solid media and streaked with bacteriological loop. The used media were sheep blood agar (Neogen, USA), brilliant green phenol red lactose sucrose agar (BPLS; Merck, Germany) and nutrient agar (Oxoid, UK); the streaked cultures were incubated for 37 °C overnight. Pure colonies were obtained by subculturing on sheep blood agar followed by identification with MALDI-TOF (see Section 2.2). Hemolysis was tested by growth with the same conditions on blood agar. The list of strains with their origin and the results of their preliminary characterization are presented in Table 1. For DNA isolation, strains were grown in 150 μL volume of lysogeny broth (LB) in 96-well plates and grown overnight at 37 °C as well. For long-term storage, overnight liquid LB cultures of the strains were supplemented with 30 *v*/*v*% sterile glycerol and stored at −70 °C. Hypermucoviscous (hmv) phenotype was demonstrated using the ‘string test’ [16] by pulling mucous string from agar-grown colonies of the respective strains.

In addition to the strains of camel calf origin, *E. coli* reference strains were used as controls for PCR reactions; these are listed in Table 2.

### 2.2. MALDI-TOF

Species-level identification of the strains was performed using mass spectrometry with the Bruker MALDI-TOF system (Bruker, Millerica, MA, USA) according to the manufacturer’s instructions. Briefly, cultures underwent a preparatory extraction with formic acid, and then they were analyzed with the instrument. Identification was conducted using the MALDI Biotyper database (Bruker, Millerica, MA, USA).

### 2.3. Determination of Antibiotic Resistance and Minimum Inhibitory Concentration (MIC) Values

MIC values for a set of thirteen antibiotics were determined using the standard method of serial dilution in Mueller–Hinton broth, according to the standard protocol of CLSI for bacterial isolates of animal origin [22]. The applied antibiotics, together with the results, are summarized in Table 3.

### 2.4. DNA Isolation and PCR-Based Investigations

The presence of significant VGs associated with pathogenic *E. coli* was checked by PCR using the primers listed in Table A1. Phylogenetic relations were mapped by the extended Clermont PCR-scheme [23].

The presence of MGEs was checked by two schemes. The Sakai prophage (Sp) typing utilizes the marker genes of twelve prophages carried by the enterohemorrhagic *E. coli* (EHEC) O157:H7 Sakai strain, described earlier [24], and the DNA isolated from this prototypic strain was used as the control (Table 2). The intact or occupied state of the integration sites of typical UPEC PAIs was also checked with targeted PCR using the primers listed in Table A1, with the strains listed in Table 2 used as controls for the reactions indicated.

For the reactions, cultures of strains grown either on agar plates or in LB broth were taken and suspended or diluted in sterile DNAse-free distilled water, then DNA was isolated by boiling the suspensions. All PCRs were performed using DreamTaq Green Mastermix (ThermoFisher, Waltham, MA, USA) per the manufacturer’s instructions. The usual heat profile of the reactions was an initial denaturation at 94 °C for 3 min, then 30 cycles of a denaturation at 94 °C for 30 s, annelation for 30 s on the optimal temperature of the primer pair used, then extension for 1 min at 72 °C. After the last cycle, a final elongation step at 72 °C for 5 min followed. In the case of the Clermont phylotyping, the fast heat profile was used [25]. Results of the reactions were visualized by agarose gel electrophoresis. All the primers used in the study are listed in Table A1.

## 3. Results

### 3.1. Species Determination and Phenotypic Traits

**Figure 1:**
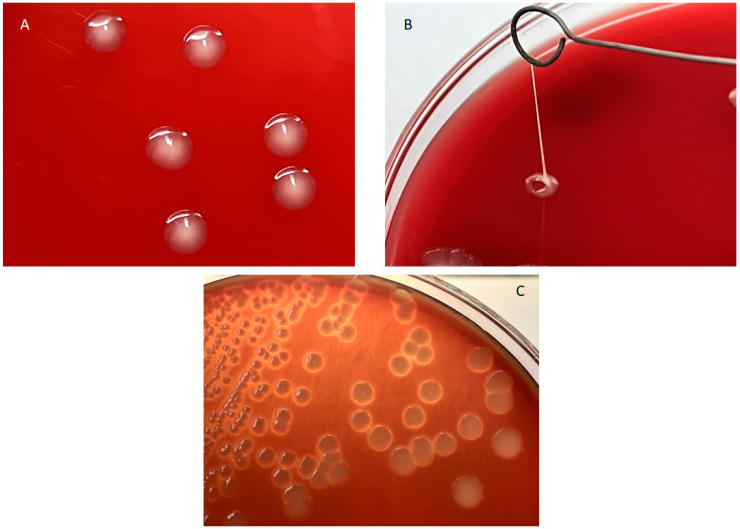
Characteristic morphologies shown by the *E. coli* strains of camel origin investigated in the study. (**A**): String test of strain TE23 showing hmv phenotype. (**B**): Mucoid but not adherent morphotype shown by strain TE11. (**C**): Hemolysis shown by strain TE14.

All 30 strains isolated from the parenchymatic organs of the dead septicaemic camel calves proved to be a member of the *E. coli* species, according to the MALDI-TOF analysis. Six strains indicated in Table 1 showed the hmv phenotype as, in their case, it was possible to drag a mucous filament > 5 mm in length from the colonies grown on agar plates (Figure 1A), and it was considerably harder to remove them from the agar surface when compared to the colonies of the other strains (‘sticky’ phenotype). Strain TE11 showed a mucous, but not hmv colony morphology (Figure 1B), while TE10 showed a sticky but not mucous phenotype. Six strains showed hemolysis (Table 1 and Figure 1C).

### 3.2. Antimicrobial Resistance Patterns and MIC Values

To test the AMR repertoire of the strains, the MIC of the strains against the thirteen antibiotics frequently applied against *E. coli* was tested; the results are shown in Table 3. Notably, there were seven strains with a MIC value higher than the MIC50 for three or more antibiotics. Three strains showed a MIC against florfenicol higher than the MIC90, and two strains, TE19 and TE20, showed a MIC against colistin higher than the MIC90 value for this antibiotic in the set. In the case of gentamycin, the strains formed two groups according to susceptibility; there were eight strains with at least MIC = 16, while the rest of the strains showed a maximum MIC value of 2 against this antibiotic.

### 3.3. Virulence Genes

We investigated the presence of known key VGs of ExPEC, as well as those of the significant intestinal pathotypes of *E. coli*. The results are summarized in Table 4. An interesting finding was that strain TE16 harbored the *eae* gene encoding the adhesin intimin, therefore belonging to the enteropathogenic *E. coli* (EPEC) pathotype. TE14, TE15 and TE18 were positive for the gene encoding cytotoxic necrotizing factor 1 (*cnf1*) and S fimbriae (*sfa*).

### 3.4. Occupied States of PAI Integration Sites

To screen for the presence of characteristic UPEC PAIs, the intact or occupied state of their characteristic integration sites in the strains’ genomes was checked using the *E. coli* K-12 derivate strain MG1655 as the positive control and the prototypic UPEC 536 strain as the internal negative control, as this strain harbors a PAI in all the investigated genomic sites; therefore, it gives a negative result for all the reactions. The results are shown in Table 4. All strains had at least three of the investigated sites disrupted, showing no product for the respective reactions. The *thrW* and *leuX* sites were disrupted in all strains.

### 3.5. Phylogenetic Relations

The phylogenetic grouping of the strains according to the extended Clermont-scheme, which is a triplex PCR reaction with supplementary reactions and checks for the presence/absence of housekeeping marker genes, is shown in Table 4. Out of the seven possible groups, all strains belonged to either group A, B1 or D. No strain proved to be a member of groups B2, C, E or F.

The results of the Sp typing based on the prophages of the EHEC O157:H7 Sakai strain are shown in Table 4. Half of the strains carried at least one of the prophage-related marker gene originally identified in the Sakai strain, suggesting that they harbor at least one corresponding or similar prophage. The majority of the other strains carried one or two prophage regions. Strain TE16 represented type 7767, indicating the carriage of four prophage regions.

## 4. Discussion

In this study, we performed phenotypic and genotypic characterization of 30 *E. coli* strains isolated from cases of sepsis in young camel calves. As all strains were isolated from extraintestinal sites where commensal *E. coli* does not occur naturally, it can be reasonably supposed that the isolated strains were the causative agents of septicaemia. The source of the strains was most likely the farm environment, with the infection occurring through the peroral route.

Six of the characterized strains displayed the hmv phenotype. While being a frequent trait of hypervirulent *Klebsiella pneumoniae* strains [26], it is rarely reported from *E. coli.* In all reported cases so far, the strains were ExPEC [16,27,28,29]. One study speculates that this trait could be a frequent, but overlooked, characteristic of UPEC strains [16]. In contrast to *K. pneumoniae*, where genes responsible for the hmv phenotype have been identified [26], the genetic background of this trait is yet to be investigated and described in *E. coli.* The hmv phenotype is frequently associated with hypervirulence in *K. pneumoniae*, in the case of which the hmv strains seem to be more capable of causing abscesses and spreading metastatically [30], suggesting that this phenotype deserves attention when characterizing and treating ExPEC infections in humans and animals.

The strains showed diverse patterns of AMR. Seven strains were potentially multidrug resistant (MDR), with one such strain and one non-MDR strain showing colistin resistance as well. Eight strains showed high resistance against gentamycin, while the rest of the strains had MIC values several dilutions lower, leading to a considerable difference between MIC50 and MIC90 for this antibiotic in the strain set. Although it is not conclusively proven [31], it is very likely that food-producing animals play an important role in the dissemination of ARGs [32], and our results corroborate this notion. The strains showed almost universally high MIC values for beta-lactams, which is consistent with earlier reports of ESBL-resistant *E. coli* being carried by camels [13,33], with one study suggesting the reservoir role of this animal in carrying potentially zoonotic ESBL-resistant *E. coli* [34]. The gene responsible for colistin resistance was reported from the *E. coli* strains of camel intestinal origin [35], and this is the first time that ExPEC strains from this animal also showed resistance against this antibiotic. The worldwide emergence of colistin-resistant strains of animal origin is a worrying trend, as colistin is considered a last-line antibiotic [36].

Three of the strains (TE14, TE15 and TE18) harbored the cytotoxic necrotizing factor type 1 (CNF-1) [37] and S fimbriae [38,39] characteristic of the subset of ExPEC referred to as necrotoxigenic *E. coli* type 1 (NTEC1) strains. Albeit sometimes reported from diarrheal cases, they are mostly associated with extraintestinal and urinary infections in both humans and animals [40,41]; *cnf+* strains have already been reported from diarrheic camels as well [12]. Only strain TE24 harbored the *papC*-encoding pyelonephritis associated pilus, an adhesion factor characteristic of UPEC [42]. There were 25 strains which were negative for all investigated VGs. Nevertheless, the site of their isolation and the pathological findings of the animals indicate the pathogenicity of the strains.

Strain TE16 carried the *eae* gene, encoding intimin, which is responsible for the intimate attachment of EPEC and EHEC cells to the host intestinal epithelium, and the characteristic pedestal formation by actin filaments in the affected epithelial cells (reviewed by [43]). The carriage of this gene classifies strain TE16 as EPEC, which is an interesting finding, as it was, similarly to other strains in the study, isolated from the parenchymatic organs of an affected animal, which died in colisepticaemia. EPEC strains are a significant intestinal pathotype affecting humans [44,45], which are frequently isolated from domestic animals, and as such, are considered zoonotic pathogens [46]. As mentioned in Section 3.5, strain TE16 also proved to be the most ‘prophage-rich’ strain with the Sp typing scheme, which could be explained by the fact that the scheme was developed for the prophages of a strain representing an intestinal pathotype. The presence of EPEC strains in the feces of both healthy and diarrheic camels was reported earlier [12,47], and was also isolated from camel carcasses and meat [14,15]. The isolation of this strain from a septic case underlines the notion that intestinal pathogenic *E. coli* and ExPEC cannot always be strictly differentiated [5], as well as that ExPEC strains in some cases may be opportunistic pathogens [48]. This difficulty of differentiation was even more underlined by the results of the phylogenetic grouping of the strains. To our surprise, no strain belonged to the B2 phylogenetic group, which is otherwise overrepresented among ExPEC strains [23] and includes almost all NTEC1 strains [49], while our *cnf1+* strains belonged to group B1.

The results of the Sp typing suggested heterogeneity in the prophage content of the strains, with ten strains containing several prophage-associated regions in multiple patterns. The investigation of characteristic UPEC PAI integration sites showed that all strains had at least three of the sites disrupted, potentially containing MGEs encoding ARGs or VGs such as *cnf1* or *papC*. The strains showed various patterns of disruption, with very few cases of repeating patterns, further pointing to the genetic variability of the isolates.

The isolation of *E. coli* strains carrying the ARGs and VGs detailed above is a cause for public health concern, as the camel calves from which they originated were raised on farms involved in camel milk production, and the consumption of unpasteurized camel milk is a widespread practice in the UAE [50]. The high proportion of antibiotic-resistant and MDR strains could be the result of the widespread use of antibiotics [51,52], and suggests that the key to an effective defense against these strains lies in herd-specific autogenous vaccines or alternative agents, such as bacteriophage-based biocontrol [53], while simultaneously reducing the use of antibiotics.

The whole genome sequencing (WGS) of the strains is currently under way. It could provide us with useful information on the content of the disrupted PAI-integration sites, heretofore unaccounted or unknown VGs or ARGs, as well as previously uncharacterized MGEs. Comparative genomic analysis with isolates from different sources should also provide useful insights on the zoonotic potential of these strains, as well as similar strains to be isolated from camels in the future.

## 5. Conclusions

This is the first report of ExPEC isolated from camel calves, showing a range of antibiotic resistance, with a diverse array of VGs and potential MGEs. The determination of WGS will provide more in-depth genomic information, especially regarding the VG and ARG repertoire, as well as MGEs including PAIs, prophages and plasmids. The unique importance of the camel as a domestic animal in the Middle East underlines the importance of exploring the pathogenic bacteria infecting them to prevent economic loss, the dissemination of antibiotic resistance and potentially zoonotic infections. Because of the genetic variability and plasticity of pathogenic *E. coli*, the close monitoring and frequent sampling of camel herds would be beneficial in order to explore emerging and potentially dangerous clones.

## Figures and Tables

**Table 1:** List of origin and phenotypical characteristics of the strains in the study; hmv: hypermucoviscous.

Strain	Pathological Findings	Source (Organ)	Phenotype	Farm
TE1	meningitis	brain	hmv	F2
TE2	septicaemia	brain		F1
TE3	septicaemia	lung		F1
TE4	septicaemia	liver		F1
TE5	septicaemia, meningitis	brain	hmv	F1
TE6	septicaemia	liver		F1
TE7	meningeal odema	brain	hmv	F1
TE8	meningitis	brain		F2
TE9	meningitis	brain		F1
TE10	meningitis	brain		F1
TE11	septicaemia, meningitis, arthritis	brain	mucuous, but not hmv	F1
TE12	septicaemia, meningitis, arthritis	brain		F2
TE13	septicaemia, meningitis	brain		F1
TE14	coccidiosis	liver		F2
TE15	stillbirth	liver		F2
TE16	septicaemia	liver		F2
TE17	septicaemia, meningitis	brain		F1
TE18	bronchopneumonia	lung		F2
TE19	septicaemia, bronchopneumonia	lung	sticky, but not hmv	F2
TE20	meningitis	brain		F2
TE21	septicaemia	brain	hmv	F2
TE22	septicaemia, bronchopneumonia, meningitis	brain	hmv	F1
TE23	septicaemia, pneumonia	lung		F1
TE24	septicaemia, meningitis	brain		F1
TE25	septicaemia, bronchopneumonia, meningitis, arthritis	brain		F2
TE26	septicaemia, bronchopneumonia, meningitis	brain		F2
TE27	septicaemia	liver		F1
TE28	septicaemia, meningitis	brain		F2
TE29	septicaemia	lung	hmv	F1
TE30	septicaemia, meningitis	brain		F2

**Table 2:** List of reference strains used as positive or internal negative controls in PCR reactions for the detection of VGs. For the primers, see Table A1.

Strain	Virulence Genes	Reference
EHEC O157:H7 Sakai	*stx1*, *stx2*, *eaeA*, Sp1-Sp17	[17]
28C	*chuA*, *yjaA*, TspE4.C2, *arpA*, *cnf1, papC*, *sfa*	[18]
MG1655	*asnT*, *pheV*, *serX, thrW, selC*, *leuX*, *chuA*, *waaC*, *argW*	[19]
UPEC 536 *	*asnT*, *pheV*, *serX*, *thrW*, *selC*, *leuX*, *chuA*, *waaC*, *argW*	[20]
1404	*cnf2*	[21]

The symbol * denotes the strain used as the internal negative control in the respective reactions.

**Table 3:** MIC values of the studied strains for different antimicrobials. Values are given in μg/mL. Abbreviations: AM: ampicillin; AMCL amoxicillin + clavulanic acid; CEFT: cefotaxime; CEFQ: cefquinome; GEN: gentamicin; NEO: neomycin; OTC: oxytetracycline; DOX: doxycycline; FLO: florfenicol; COL: colistin; ENR: enrofloxacin; MAR: marbofloxacin; TMP-SMX: trimethoprim + sulfamethoxazole.

	Antibiotics												
Strain	AM	AMCL	CEFT	CEFQ	GEN	NEO	OTC	DOX	FLO	COL	ENR	MAR	TMP-SMX
	Minimal Inhibitory Concentrations (MIC values)												
**TE1**	128	32	16	16	1	4	128	32	8	0.125	0.015	0.015	256
**TE2**	128	64	32	32	64	2	128	32	8	0.25	0.015	0.015	256
**TE3**	128	32	32	32	64	64	128	64	128	0.25	32	32	256
**TE4**	128	128	32	8	2	4	128	64	16	0.25	32	16	256
**TE5**	128	128	32	32	2	4	128	16	8	0.25	32	32	256
**TE6**	128	128	32	32	64	2	128	64	8	0.25	32	16	256
**TE7**	128	32	32	32	64	2	8	4	128	0.25	32	16	256
**TE8**	128	32	32	32	2	64	128	64	8	0.125	32	8	256
**TE9**	128	32	32	32	16	64	128	64	8	0.125	32	16	256
**TE10**	128	16	16	16	2	4	8	4	8	0.125	32	16	256
**TE11**	128	128	32	32	0.5	4	16	8	8	0.125	32	32	256
**TE12**	128	32	32	32	2	64	128	64	8	0.125	32	16	256
**TE13**	128	32	16	16	2	4	128	16	8	0.125	0.015	0.015	256
**TE14**	128	32	32	32	2	64	128	64	8	0.125	32	8	256
**TE15**	128	16	32	32	2	64	128	64	8	0.125	32	8	256
**TE16**	128	32	32	32	1	64	4	32	8	0.125	0.06	0.015	256
**TE17**	128	32	32	32	64	64	128	64	128	0.125	32	32	256
**TE18**	128	64	32	32	1	64	128	64	8	0.25	32	16	256
**TE19**	128	64	32	32	2	64	128	64	8	0.5	32	16	256
**TE20**	128	32	16	4	2	2	128	16	8	0.5	32	16	256
**TE21**	8	8	16	4	2	4	4	4	8	0.25	0.015	0.015	256
**TE22**	8	8	16	4	2	4	8	8	8	0.25	32	32	256
**TE23**	128	32	16	4	64	64	128	64	8	0.25	32	32	256
**TE24**	8	8	16	4	1	2	8	4	8	0.25	32	32	256
**TE25**	128	32	32	32	16	64	128	64	8	0.25	32	16	256
**TE26**	128	64	32	2	2	64	128	32	8	0.25	32	8	256
**TE27**	128	32	16	2	2	64	128	16	8	0.25	0.015	0.015	256
**TE28**	128	32	16	2	2	64	128	16	8	0.25	0.015	0.015	256
**TE29**	128	64	8	2	2	64	128	64	16	0.25	32	32	256
**TE30**	128	64	8	2	2	64	8	8	16	0.25	32	16	256
**MIC_50_**	128	32	32	32	2	64	128	32	8	0.25	32	16	256
**MIC_90_**	128	128	32	32	64	64	128	64	16	0.25	32	32	256

**Table 4:** Carriage of VGs, state of UPEC PAI integration sites, and phylogenetic grouping of the strains. The results of the Sp typing are shown as a four-digit code, as described in [24]. The intact or disrupted state of UPEC PAI integration sites is shown as: ‘i’: intact, ‘d’: disrupted.

	Virulence Genes								Phylogenetic Group	Intact/Disrupted State of UPEC PAI Integration Sites										Prophage Type
Strain	*stx1*	*stx2*	*eae*	*cdt*	*cnf1*	*cnf2*	*papC*	*sfa*		*asnT*	*pheV*	*serX*	*thrW*	*selC*	*leuX*	*fimZ*	*chu*	*waa*	Number of Disrupted PAI Integration Sites	
TE1	-	-	-	-	-	-	-	-	A	d	d	i *	d	i	d	d	i	i	5	8888
TE2	-	-	-	-	-	-	-	-	A	d	d	i *	d	d	d	d	i	i	6	8888
TE3	-	-	-	-	-	-	-	-	B1	d	d	d	d	i	d	d	i	i	6	8858
TE4	-	-	-	-	-	-	-	-	A	d	d	i	d	d	d	d	i	i	6	8888
TE5	-	-	-	-	-	-	-	-	A	d	d	i *	d	d	d	d	i	i	6	8857
TE6	-	-	-	-	-	-	-	-	B1	d	d	d	d	d	d	d	i	i	7	5868
TE7	-	-	-	-	-	-	-	-	B1	d	d	d	d	i	d	i	i	i	5	8788
TE8	-	-	-	-	-	-	-	-	B1	d	d	d	d	i	d	d	i	i	6	8888
TE9	-	-	-	-	-	-	-	-	B1	d	d	d	d	d	d	i	i	i	6	8888
TE10	-	-	-	-	-	-	-	-	A	d	d	i *	d	d	d	d	i	i	6	8888
TE11	-	-	-	-	-	-	-	-	A	d	i	i *	d	d	d	i	i	i	4	8858
TE12	-	-	-	-	-	-	-	-	B1	d	d	d	d	i	d	i	i	i	5	8888
TE13	-	-	-	-	-	-	-	-	A	d	i	i *	d	d	d	i	i	i	4	8888
TE14	-	-	-	-	+	-	-	+	B1	d	d	i *	d	i	d	d	i	i	5	8888
TE15	-	-	-	-	+	-	-	+	B1	d	d	d	d	i	d	d	i	i	6	8888
TE16	-	-	+	-	-	-	-	-	D	i	d	i *	d	d	d	d	i **	i	5	7767
TE17	-	-	-	-	-	-	-	-	A	d	d	i *	d	d	d	d	i	i	6	8857
TE18	-	-	-	-	+	-	-	+	B1	d	d	d	d	i	d	i	i	i	5	8888
TE19	-	-	-	-	-	-	-	-	B1	d	d	d	d	i	d	d	i	i	6	8888
TE20	-	-	-	-	-	-	-	-	A	d	d	i *	d	d	d	d	i	i	6	8858
TE21	-	-	-	-	-	-	-	-	D	i	d	i *	d	i	d	d	i **	i	4	8787
TE22	-	-	-	-	-	-	-	-	A	d	i	i	d	d	d	i	i	i	4	8828
TE23	-	-	-	-	-	-	-	-	A	d	i	i	d	d	d	i	i	i	4	8828
TE24	-	-	-	-	-	-	+	-	A	d	d	i *	d	d	d	d	i	i	6	8838
TE25	-	-	-	-	-	-	-	-	B1	d	d	d	d	d	d	d	i	i	7	8888
TE26	-	-	-	-	-	-	-	-	B1	d	d	d	d	i	d	i	i	i	5	8888
TE27	-	-	-	-	-	-	-	-	B1	d	d	d	d	i	d	i	i	i	5	8888
TE28	-	-	-	-	-	-	-	-	A	d?	i	i	d	d	d	i	i	i	3	8828
TE29	-	-	-	-	-	-	-	-	A	d	i	i	d	i	d	i	i	i	3	8828
TE30	-	-	-	-	-	-	-	-	A	d	d	i *	d	d	d	d	i	i	6	8788

The symbol * denotes the length of the product different from the one expected. The symbol ** denotes a weak product.

## Data Availability

All data related to the research are included in the article.

## Appendix A

**Table A1:** List of primers for PCR reactions used in the study. Targets with more than two primers were detected with multiplex reactions. Primers taken from ref. [54] encode tRNA genes. Sp1-17 refer to the prophage genes of the EHEC O157:H7 Sakai strain. Their primers were grouped into triplex reactions, as indicated (for details see ref. [24]). Abbreviations of pathotypes: STEC: Shiga toxigenic *E. coli;* EPEC: enteropathogenic *E. coli*; NTEC: necrotoxigenic *E. coli*.

Target Gene/Region	Related Pathotype	Primer Name	Sequence 5′ -> 3′	Annealing Temperature (°C)	Reference
*stx1*	STEC, EHEC	stx1-det-F1	GTACGGGGATGCAGATAAATCGC	52	[55]
		stx1-det-R1	AGCAGTCATTACATAAGAACGYCCACT	52	[55]
*stx2*	STEC, EHEC	F4	GGCACTGTCTGAAACTGCTCCTGT	52	[55]
		R1	ATTAAACTGCACTTCAGCAAATCC	52	[55]
		F4-f	CGCTGTCTGAGGCATCTCCGCT	52	[55]
		R1-e/f	TAAACTTCACCTGGGCAAAGCC	52	[55]
*eaeA*	EPEC	B52	AGGCTTCGTCACAGTTG	52	[56]
		B53	CCATCGTCACCAGAGGA	52	[56]
*cdtB*	various	CDT-s1	GAAAGTAAATGGAATATAAATGTCCG	55	[57]
		CDT-s2	GAAAATAAATGGAACACACATGTCCG	55	[57]
		CDT-as1	AAATCACCAAGAATCATCCAGTTA	55	[57]
		CDT-as2	AAATCTCCTGCAATCATCCAGTTA	55	[57]
*cnf1*	NTEC	CNF1-A	GAACTTATTAAGGATAGT	50	[58]
		CNF1-B	CATTATTTATAACGCTG	50	[58]
*cnf2*	NTEC	CNF1-A	AATCTAATTAAAGAGAAC	50	[58]
		CNF2-B	CATGCTTTGTATATCTA	50	[58]
*papC*	ExPEC	pap1	GACGGCTGTACTGCAGGGTGTGGCG	65	[59]
		pap2	ATATCCTTTCTGCAGGGATGCAATA	65	[59]
*sfa*	ExPEC	sfa1	CTCCGGAGAACTGGGTGCATCTTAC	65	[59]
		sfa2	CGGAGGAGTAATTACAAACCTGGCA	65	[59]
*chuA*		ChuA.1	GACGAACCAACGGTCAGGAT	59	[25]
		ChuA.2	TGCCGCCAGTACCAAAGACA	59	[25]
*yjaA*		YjaA.1	TGAAGTGTCAGGAGACGCTG	59	[25]
		YjaA.2	ATGGAGAATGCGTTCCTCAAC	59	[25]
TspE4.C2		TspE4C2.1	GAGTAATGTCGGGGCATTCA	59	[25]
		TspE4C2.2	CGCGCCAACAAAGTATTACG	59	[25]
*arpA* group E		ArpAgpE.f	GATTCCATCTTGTCAAAATATGCC	59	[60]
		ArpAgpE.r	GAAAAGAAAAAGAATTCCCAAGAG	59	[60]
*arpA* group C		trpAgpC.1	AGTTTTATGCCCAGTGCGAG	59	[60]
		trpAgpC.2	TCTGCGCCGGTCACGCCC	59	[60]
*asnT*		asnT1	TATTCGCCCCGTTCACACG	60	[54]
		asnT2	GATACCCGCAGTTAAGCGG	60	[54]
*pheV*		pheV1	ACGAGACGAGGCGAATCAG	60	[54]
		pheV2	CCGTTGAGCGAACGGATTG	60	[54]
*serX*		serX1	CTCTCTTGCGCATTTTGATTG	60	[54]
		serX2	AGACAGGAACGACAATTTGG	60	[54]
*thrW*		thrW1	AAGGCCATTGACGCATCGC	60	[54]
		thrW2	CCATTTACCCCACTCAGCG	60	[54]
*selC*		selC1	GCGTGTATTAGGCGGAAAAAAC	60	[54]
		selC2	CCCTGAACTTCCCCACAAC	60	[54]
*leuX*		leuX1	GTGGCGTGCGACAGGTATA	60	[54]
		leuX2	GTTTCTCCGGCCCTAAGAC	60	[54]
*chuA*		chu1	GGTATTTATGGTTCAGTGATG	60	[54]
		chu4	TTTTCTCACTCAAATTGAACG	60	[54]
*waaC*		waalücke 1	CGCACTCACTGATGCCCAGCA	60	[54]
		waalücke 2	AGTCCAATCCATGCTTTACGCCAT	60	[54]
Sp1 (reaction 1)		31.1-f	CGCCAGCTAAATCGAACCGCAT	68	[24]
		31.1-r	CGGCTGATGATGACGACTTACTG	68	[24]
Sp3 (reaction 1)		84.3-f	CAGCAGATTGAAGCAGCACTCG	68	[24]
		84.3-r	GAATAAGAGCTGAGTCGTGCGG	68	[24]
Sp4 (reaction 1)		106.5-f	ACGATTGAGCTGACACCGGGC	68	[24]
		106.5-r	CCGGGCTTAATGTGCGGGCC	68	[24]
Sp5 (reaction 2)		110.2-f	CGAAGGGGCAACCGCGAAAATA	68	[24]
		110.2-r	CCCTTGTTACTTTCAGCATTCCG	68	[24]
Sp6 (reaction 2)		122.1-f	GGTGATGGTTTGTGGGAGAGGT	68	[24]
		122.1-r	TTGGGGGCTTAACGAATACCCC	68	[24]
Sp8 (reaction 2)		124.3-f	GCGTGCAGGTAATGGTAATCCG	68	[24]
		124.3-r	TTTAATGCCGTCCTGTTCCTGAGA	68	[24]
Sp10 (reaction 3)		145.3-f	TCCGGCATTTTCCCTGACACCA	68	[24]
		145.3-r	TATCGCGTGCCTCCTGGGTTAT	68	[24]
Sp9 (reaction 3)		133.1-f	GGCATCTAACGGTCTGGTGCC	68	[24]
		133.1-r	CAGCAGAAGCGAACAGCCGTCT	68	[24]
Sp11 (reaction 3)		164.1-f	TGACATCCACCACATCCGCAGAA	68	[24]
		164.1-r	TGTGAGGAAGAGCAGACGGAGA	68	[24]
Sp15 (reaction 4)		220.4-f	TACAGCGAATGCCAAATACGCTC	68	[24]
		220.4-r	TCACCCCTACAGAGAGCAAAAGAG	68	[24]
Sp14 (reaction 4)		204.4-f	CCAAAATACATCCACCCACCGCA	68	[24]
		204.4-r	AACGCATAGAAGAGCTGGAGGC	68	[24]
Sp17 (reaction 4)		276.1-f	CAGGTGGGTTGGGTAAGGTTTG	68	[24]
		276.1-r	GATGGCTGCTATGGGGATGGC	68	[24]
